# Betalains and their applications in food: The current state of processing, stability and future opportunities in the industry

**DOI:** 10.1016/j.fochms.2022.100089

**Published:** 2022-02-21

**Authors:** S.J. Calva-Estrada, M. Jiménez-Fernández, E. Lugo-Cervantes

**Affiliations:** a Unidad de Tecnología Alimentaria, Centro de Investigación y Asistencia en Tecnología y Diseño del Estado de Jalisco (CIATEJ) A.C., Camino Arenero 1227, El Bajío, Zapopan, Jalisco C.P. 45019, Mexico; b Centro de Investigación y Desarrollo en Alimentos, Universidad Veracruzana, Av. Doctor Luis Castelazo, Industrial Las Animas, Xalapa Enríquez, Veracruz C.P. 91190, Mexico

**Keywords:** Alkaloid, Betalamic acid, Color, Encapsulation, Stability, Pigment, Wall material

## Abstract

Betalains are water-soluble nitrogenous pigments with coloring properties and antioxidant activities, which is why they have been incorporated into several foods. However, their use is limited by their instability in response to different factors, such as, pH, oxygen, water activity, light, metals, among others. In this work, a review of up-to-date and relevant information is presented on the primary natural sources of betalains. Additionally, the advantages and disadvantages of the primary betalain extraction techniques are discussed and compared. The results of these studies were focused on the stability of betalains when incorporated into foods, either in pure or encapsulated form, and they are discussed through different technologies. Lastly, the most relevant information related to their stability and a projection of their promising future applications within the food industry is presented.

## Highlights

• Several fruits, plants, and roots are sources of betalains.
• Betalains analysis can be performed by spectroscopic and chromatographic methods.
• Betalains extraction techniques have advantages and disadvantages.
• Betalains encapsulation increases its stability and possibility of incorporation in foods.
• The incorporation of betalains in food packaging is a potential application.

## 1 Introduction

Interest in incorporating natural additives, such as colorants and bioactive compounds into the food industry has increased in recent years, favoring the attention towards the nutritional value and sensory attributes of the products as well as improving food safety (Kanatt, 2020, Zin et al., 2020a). Given this concern, betalains constitute a group of compounds with great potential for the enrichment and supplementation of foods due to their pigmentation, antioxidant, antimicrobial properties, and other bioactivities associated with putative health benefits for humans (Prieto-Santiago et al., 2020, Zin et al., 2020b). However, the application of betalains as antioxidants and/or natural colorants in industrialized products is associated with challenges related to maintaining their chemical stability and, therefore, bioactivities and value as pigments (Castro-Enríquez et al., 2020). In this context, the present work provides a compilation of the most relevant and up to date findings that allow us to understand the chemical properties of betalains and the factors associated with their stability. We address the natural sources studied to date from which this group of pigments can be obtained, and we analyze the extraction and analysis methods applied to optimize and obtain these compounds more efficiently for future industrial applications. Lastly, the potential incorporation of betalains into foods to improve their acceptability and shelf life is discussed, to address the effect of the matrix in which each compound is incorporated. The effects of the storage conditions on the conservation of the betalain antioxidant and pigmentation capacity as well as potential projections and new application trends of these compounds in the food industry are noted.

## 2 Chemistry and natural sources of betalains

**Fig. 1:**
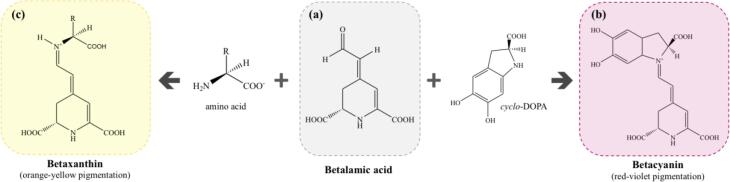
(a) Betalamic acid, the basic structure of the betalains; (b) general structure of the betacyanins derived from the condensation of the betalamic acid with *cyclo*-DOPA; and (c) general structure of the betaxanthines derived from the condensation of the betalamic acid with amino acids or its derivatives.

Betalains are nitrogenous pigments derived from betalamic acid (4-(2-oxoethylidene)-1,2,3,4-tetrahydropyridine-2,6-dicarboxylic acid) (Fig. 1a), constituting the basic structure of betalains (Slimen et al., 2017). Betalains have high hydrophilicity due to the hydroxyl groups (–OH) on their structures, and these groups lead to charge polarization and the formation of hydrogen bonds responsible for this property (Fathordoobady et al., 2016). From the basic structure of betalamic acid, condensations with different molecules are generated that originate from the two structural classifications of betalains. The first group is made up of structures in which betalamic acid is condensed by *cyclo*-DOPA (*cyclo*-L-3,4-dihydroxyphenylalanine) or its glucosyl derivatives, which are known as betacyanins (Fig. 1b). The second classification corresponds to betaxanthins (Fig. 1c), which originate from the condensation of betalamic acid with amino compounds (amino acids, amines, or derivatives) (Silva et al., 2020, Zin et al., 2020b).

**Fig. 2:**
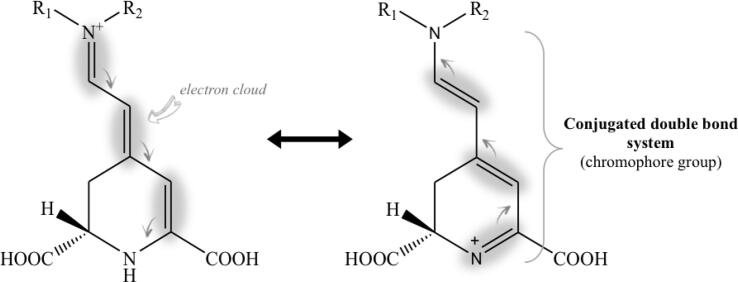
Resonance structure of betalain. The gray shade represents the electron cloud within the conjugated double bond system, and the gray arrows indicate the conjugate displacement of electron clouds.

The type of substitution linked to the basic structure of betalain has a strong impact on its primary characteristic, its pigmentation, a property associated with the resonance between the electrons of the conjugated double bond system in the structure (Fig. 2). This structure acts as a chromophore with the absorption of visible light in the 457–485 nm region, producing the characteristic orange–yellow color of betaxanthins. For betacyanins, the substitution of the basic structure by an aromatic nucleus (such as *cyclo*-dopa) extends the electronic resonance system, producing a bathochromic shift of 50 to 70 nm, so its characteristic absorption is close to 532–550 nm, conferring a red-violet pigmentation (Choo, 2019, Rodriguez-Amaya, 2019).

Betalains are secondary nitrogenous metabolites present in the seeds, fruits, flowers, leaves, stems, and roots of the *Amaranthaceae*, *Cactaceae* and *Chenopodiaceae* families to which they confer their characteristic red-yellow pigmentation, as well as multiple properties, including antioxidant, anti-cancer, antilipidemic, and antimicrobial activity (Gengatharan et al., 2016, Hu et al., 2020, Otálora et al., 2019). Therefore, a search was performed for plants that have been shown to contain this type of pigment. Table 1 provides a summary of the primary findings concerning the types and parts of these plants. The betalains content and the primary antioxidant properties exhibited by these pigments are given below. In the flowers/bracts of *Amaranthus* spp., betalain concentrations between 0.95 and 6.02 mg/100 g have been found (Li et al., 2015), and in *Bougainvillea* spp. 465 mg of betacyanins and 116 mg of betaxanthines/100 g show inhibition of the radicals ABTS and DPPH of 72.68 and 61.24%, respectively (Orozco-Villafuerte et al., 2019).

**Table 1:** Summary of natural sources of betalains and their reported concentrations.

**Sample**	**Extraction method**	**Betalains**	**Betacyanins**	**Betanines**	**Amaranthins**	**Betaxanthins**	**Reference**
		(mg/100 g)	(mg/100 g)	(mg/100 g)	(mg/100 g)	(mg/100 g)	
***Flowers/Bracts***							
*Amaranthus* spp.	Solid-liquid extraction	0.95–6.02	–	–	0.45–2.76	–	Li et al., 2015
*Bougainvillea spectabilis*	Solid-liquid extraction	–	465	–	–	116	Orozco-Villafuerte et al., 2019
***Fruits***							
*Basella rubra L.*	Maceration	13.81	–	–	–	–	Kumar et al., 2020
	Solid-liquid extraction	143.76	124.18	–	–	19.16	Kumar et al., 2015
	Ultrasound-assisted extraction (UAE)	–	142	–	–	535	Maran & Priya, 2015
Prickly pear (*Optunia ficus indica*)	Solid-liquid extraction	41.54	–	–	–	–	Prakash-Maran, Manikandan, & Mekala, 2013
Prickly pear (*Opuntia* spp.)	High Pressure Carbon Dioxide (HPCD)	89	–	–	–	–	Nunes et al., 2015
	High-Pulsed Electric Fields (HPEF)	285 – 2,252	159 – 1,655	–	–	126 – 686	Jiménez-Alvarado et al., 2015
Red dragon fruit (*Hylocereus polyrhizus*)	Solid-liquid extraction	–	82.79	–	–	–	Ramli et al., 2014
	Ultrasound-assisted extraction (UAE)	–	71.34	–	–	–	Ramli et al., 2014
Red pitaya (*Stenocereus stellatus*)	Ultrasound-assisted extraction (UAE)	479.3	–	–	–	–	Pérez-Loredo et al., 2017
Xoconostle (*Opuntia joconostle*)	Solid-liquid extraction	–	92	–	–	–	Sanchez-Gonzalez et al., 2013
***Fruit peels***							
*Opuntia engelmannii*	Ultrasound-assisted extraction (UAE)	20,160	–	–	–	–	Melgar et al., 2019
	Microwave-assisted extraction (MAE)	13,290	–	–	–	–	Melgar et al., 2019
Red dragon fruit (*Hylocereus polyrhizus*)	Solid-liquid extraction	–	18.67	–	–	–	Ramli et al., 2014
	Ultrasound-assisted extraction (UAE)	–	17.64	–	–	–	Ramli et al., 2014
***Leaves***							
*Amaranthus* spp.	Solid-liquid extraction	16.90–20.93	–	–	7.75–9.67	–	Li et al., 2015
Red amaranth (*Amaranthus cruentus*)	Solid-liquid extraction	–	159.09	–	–	–	Das et al., 2019
***Roots***							
Grown red and golden beets (*Beta vulgaris* L.)	Ultrasound-assisted extraction (UAE)	–	–	3.75 – 75.64	–	–	Wang et al., 2020
Red beetroot (*Beta vulgaris* L.)	Solid-liquid extraction	–	390	–	–	214	Silva et al., 2020
	Solid-liquid extraction	–		156	–	–	Neagu & Barbu, 2014
	Solid-liquid extraction	–	30.9	–	–	16.3	Swamy et al., 2014
	Ultrasound-assisted extraction (UAE)	–	445	–	–	242	Silva et al., 2020
***Seeds***							
*Amaranthus* spp.	Solid-liquid extraction	0.07–0.96	–	–	0.04–0.44	–	Li et al., 2015
***Whole plant***							
*Alternanthera sessilis*	Solid-liquid extraction	–	–	7,310	7,270	7,450	Yap et al., 2019
***Others***							
*Amaranthus* spp. stalks	Solid-liquid extraction	0.56–1.54	–	–	0.28–0.70	–	Li et al., 2015
*Amaranthus* spp. sprouts	Solid-liquid extraction	2.69	–	–	1.28	–	Li et al., 2015
Colored quinoa (*Chenopodium quinoa* Willd) hulls	Ultrasound-assisted extraction (UAE)	–	96.47	–	–	201.01	Laqui-Vilca et al., 2018
Glasswort (*Salicornia fruticosa*) air parts	Solid-liquid extraction	12,990	–	–	–	–	Mohamed et al., 2018

Some fruits such as prickly pear (*Opuntia spp*.), red dragon fruit (*Hylocereus polyrhizus*), and xoconostle (*Opuntia joconostle*) have also been shown to be important sources of these pigments at concentrations ranging from 13.81 to 2.252 mg/100 g (Jiménez-Alvarado et al., 2015, Kumar et al., 2020, Pérez-Loredo et al., 2017, Ramli et al., 2014, Sanchez-Gonzalez et al., 2013). The roots of different varieties of the beet *Beta vulgaris* L. represent one of the most studied natural sources due to their high content of betalains, at between 30.9 and 445 mg of betacyanins/100 g and 16.3 to 242 mg of betaxanthins/100 g (Silva et al., 2020, Swamy et al., 2014). This natural source has shown a high ABTS scavenging activity of 229.83–300.76 mg of AAE/g in contents of 260–436.5 mg of betanin/100 g of fresh root (Wang et al., 2020). In addition, 0.19 mmol ET/g (by DPPH assay), 0.15 mmol ET/g (by FRAP), and 4.88 mmol ET/g (by ABTS) were found at concentrations of 4.6 mg of betacyanins and 2.6 mg of betaxanthins/g (Silva et al., 2020). The peels of red dragon fruits and prickly pears have also been reported as excellent sources of betalains, with up to 18.67 mg of betacyanins/100 g and 20,160 mg of betalains/100 g of peel, respectively. The peel of the red dragon fruits reflected an antioxidant activity (inhibition percentage, by ABTS assay) of 3.04% and a reducing power (by FRAP) of 200.83 mol Fe^2 +^/g but a low correlation with the betacyanin content (Ramli et al., 2014). In contrast to prickly pear peel, a synergistic effect was observed between the antioxidant activity of 2.4 mg/mL (IC_50_ value, by reducing power) and the betalain content (Melgar et al., 2019). These interesting results could be used to give value to fruit parts, generally considered a residue, that are used to obtain these pigments. Given these properties, other parts of the plant specimens can be used, including the leaves, stalks, sprouts, seeds, seed hulls, or the whole plant (Laqui-Vilca et al., 2018, Li et al., 2015, Mohamed et al., 2018, Yap et al., 2019). Li et al. (2015) observed a positive correlation between the betalains content of *Amaranthus* spp. and the antioxidant activity evaluated by FRAP method (0.63–62.21 mmol AAE/g) and ORAC (30.67–451.37 mmol TE/g), and concluded that the leaves of the species *Amaranthus hypochondriacus* had overwhelmingly higher antioxidant activities compared to other species and parts of the same plant, including its seeds, flowers, stems, and fruits. These findings are relevant when considering rapidly developing plant parts, such as aerial parts and leaves, as well as fruit residue fractions, such as the peel, when searching for alternative sources of betalains for use at an industrial level in an economical, sustainable, and renewable way.

## 3 Betalain extraction processes

In the interest of obtaining, studying, and evaluating the potential applicability of betalain pigments for the industry, multiple studies have been focused on the optimization of conventional extraction processes, using response surface methodology (RSM) (Kumar et al., 2017, Singh et al., 2017, Zin et al., 2020a, Zin et al., 2019) and multivariate analysis by principal components analysis (PCA) (Neagu and Barbu, 2014, Silva et al., 2020) to maximize yields and maintain their stability. The betalains extraction conditions from natural sources that have been optimized and/or suggested according to some results of the most recent studies are listed in Table 2.

**Table 2:** Summary of the optimal or suggested conditions for the extraction of betalains by conventional solid–liquid extraction from natural sources.

**Sample**	**Compound(s)**	**R_L/S_**	**Solvent**	**Solvents ratio (%)**	**Temperature**	**Time**	**Other condition**	**Reference**
*Alternanthera sessilis* (red)	Amaranthin, betaxanthin and betanin	20 mL/g	ethyl acetate	100	50 °C	24 h	stirring speed of 200 rpm	Yap et al., 2019
*Bougainvillea glabra* floral bracts	betalains (as optical density)	17 mL/g	methanol:water	25:75	22.5 °C	6 h		Kumar et al., 2017
Glasswort (*Salicornia fruticosa*) air parts	betalains	20 mL/g	ethanol:water (acidified with 0.5% citric acid)	20:80	40 °C	30 min		Mohamed et al., 2018
*Hylocereus polyrhizus* flesh	betacyanins	10 mL/g	ethanol:water	50:50	room temp.	20 min	stirring speed of 300 rpm	Fathordoobady et al., 2016
	betacyanins	10 mL/g	ethanol:water	70:30	room temp.	20 min	stirring speed of 300 rpm	Fathordoobady et al., 2016
Red amaranth (*Amaranthus cruentus*)	betacyanins	40 mL/g	water (pH 5)	100	50 °C	60 min		Das et al., 2019
Red beetroot (*Beta vulgaris* L.)	betacyanins and betaxanthins	75 mL/g	water	100	30 °C	30 min	stirring speed of 40 rpm	Silva et al., 2020
	betanin	5 mL/g	water (acidified with 0.5% citric acid and 0.1% ascorbic acid)	100	70 °C			Neagu & Barbu, 2014
	betalamic acid, betacyanin and betaxanthin	33 mL/g	water	100	60 °C	84 min		Swamy et al., 2014
Red beetroot (*Beta vulgaris* L.) peel	betacyanins and betaxanthins	1.25 mL/g	ethanol:water	15:85	20 °C	60 min	stirring speed of 215 rpm	Zin et al., 2020
Red beetroot (*Beta vulgaris*) powder	betalains	100 mL/g	ethanol:water (pH 5)	50:50	30 °C			Pandey et al., 2018
Beetroots (*Cylindra type*) peel	betacyanins and betaxanthins	10 mL/g	ethanol:water	25:75	50 °C	50 min		Zin et al., 2019
Red dragon fruit (*Hylocereus polyrhizus*) peel	betacyanin	25 mL/g	water	100	50 °C	120 min	stirring speed of 200 rpm	Ramli et al., 2014
Prickly pear (*Optunia ficus indica*) fruits	betalains	42 mL/g	water (acidified with citric acid, pH 6.9)	100	42 °C	115 min		Prakash-Maran et al., 2013
Xoconostle (*Opuntia joconostle*) fruit	betacyanin	20 mL/g	methanol:water (acidified with citric acid, pH 5)	20:80	15 °C	10 min		Sanchez-Gonzalez et al., 2013

RL/S mean: Ratio liquid (solvent)/solid.

Due to the hydrophilic nature of betalains, the methods developed for their extraction from different natural sources include the use of water, methanol- and ethanol–water mixtures in different ratios, and ethyl acetate. Water has a demonstrated efficiency at extracting betacyanins and betaxanthins from red amaranth (*Amaranthus cruentus*) (Das et al., 2019), red beet root (*Beta vulgaris* L.) (Neagu and Barbu, 2014, Silva et al., 2020, Swamy et al., 2014), red dragon fruit (*Hylocereus polyrhizus*) peel (Ramli et al., 2014), and prickly pear (*Opuntia ficus* indica) fruits (Prakash-Maran et al., 2013). Aqueous methanol solution has been found the most effective for extracting betalains from *Bougainvillea glabra* floral bracts (Kumar et al., 2017) and Xoconostle (*Opuntia joconostle*) fruit (Sanchez-Gonzalez et al., 2013). Ethanol solutions have provided a higher yield of betalains from glasswort (*Salicornia fruticosa*) (Mohamed et al., 2018), *Hylocereus polyrhizus* flesh (Fathordoobady et al., 2016), red beet root (*Beta vulgaris* L.) peel (Zin et al., 2020a) and powder (Pandey et al., 2018), and beet-roots (*Cylindra type*) peel (Zin et al., 2019). In addition, ethyl acetate is used to extract *Alternanthera sessilis* (Yap et al., 2019). It should be noted that during the selection of the appropriate solvent for extracting any phytochemical, the applicability of the final extraction product must be considered since, if it is a food additive, some options (such as methanol and ethyl acetate) are not suggested due to their potential toxicity. With this consideration in mind, solvents such as water, ethanol and a mixture of these solvents are recommended. The extraction of betacyanins with water has shown greater efficiency with respect to aqueous ethanol solutions due to the nucleophilic attack of ethanol on the aldimine bond (N = CH) of betalains originating from its degradation via decarboxylation (Das et al., 2019, Sanchez-Gonzalez et al., 2013). However, when samples with high pectin contents are used, such as red pitaya (*Hylocereus polyrhizus*) flesh and peel, a lower proportion of water in the aqueous ethanol extraction solvent is recommended to reduce mucilaginization due to the solubilization of water-soluble carbohydrates that hinder any subsequent filtration processes (Fathordoobady et al., 2016). In addition to choosing a suitable solvent, selecting the other extraction conditions has been shown to significantly improve betalains extraction efficiency, including the liquid/solid ratio, pH, temperature, and time (Kumar et al., 2017, Zin et al., 2020a). The liquid (solvent)/solid (sample) ratio (R_L/S_) recommended for conventional extraction varies over a range of 1.25–100 mL/g (Table 2) and is dependent on the type of sample. Having more sample available in the extraction mixture (solvent/sample) positively favors the betalains yield due to the more significant amount of extractable material in the medium (Prakash-Maran et al., 2013). However, a higher volume of solvent favors better hydration and the swelling of solid samples, reduces the viscosity of the medium and thus improves the extraction efficiency (Silva et al., 2020). Zin et al., (2020a) observed that the increase in R_L/S_ improved the betacyanin and betaxanthin extraction yields from red beetroot (*Beta vulgaris* L.) when using an extraction temperature of 20 °C. Thus, the R_L/S_ plays an important role when the extraction process is performed at low temperatures, in which a higher R_L/S_ ratio must be considered to increase the diffusivity of the pigment in the medium and to decrease the time to reach the final equilibrium state, which improves the extraction efficiency (Mohamed et al., 2018, Neagu and Barbu, 2014, Zin et al., 2019).

### 3.1 Effect of pH on the extraction process

The pH also plays a very important role in the betalains extraction process since it can affect compound stability and thus decrease its extraction efficiency. Neagu and Barbu, (2014) observed that the pH has a positive impact on the extraction process when performed at a low temperature (20 °C) compared with a temperature of 70 °C, at which the pH does not have a significant influence. Das et al. (2019) observed an increase in the betalains extraction yield from red amaranth (*Amaranthus cruentus*) with acidification of the medium to pH 5. Similar results have been reported in red beet-root (*Beta vulgaris* L.) powder (Pandey et al., 2018) and Xoconostle (*Opuntia joconostle*) fruit (Sanchez-Gonzalez et al., 2013). Therefore, acidifying the extraction medium is recommended to improve the accumulation of betalains and prevent their degradation (Mohamed et al., 2018). Acidifying agents such as citric acid have been commonly used because they act as neutralizing agents for the electrophilic center of betalains, which improves its stability (Prakash-Maran et al., 2013).

### 3.2 Effect of heat and time on the extraction process

The extraction temperature and time significantly affect the extraction efficiency (Silva et al., 2020, Swamy et al., 2014). According to the results summarized in Table 2, an optimal conventional extraction temperature of 20–50 °C is recommended. An extraction at a low temperature of 10 °C is not enough for the complete extraction of betalains (Pandey et al., 2018). An increase in temperature to no higher than 55 °C, improves the extraction performance due to the softening of the plant tissue and the increased permeability of the cell membrane, favoring the more significant release of pigments whose solubility and diffusion coefficient are also increased (Maran and Priya, 2016, Zin et al., 2019). The time determines the period in which the extractable matter will be in contact with the extraction agent. A longer extraction time favors the process yield (Prakash-Maran et al., 2013). The extraction time has a significant effect when temperatures above 60 °C are used during the process, leading to a reduction in the betalains content due to the hydrolytic degradation associated with prolonged thermal exposure (Silva et al., 2020). However, at lower temperatures (30–50 °C), an extraction time of greater than 115 min also leads to the degradation of these pigments. The extraction time is also influenced by the R_L/S_, in which more sample and a longer extraction time are necessary to increase the yield levels (Kumar et al., 2017). This effect occurs because by increasing the mass of solute in the solvent, the time required to reach the equilibrium of mass and heat transfer is proportionally higher (Zin et al., 2019). Lastly, the extraction time is highly variable and depends on the type and nature of the sample. For example, (Fathordoobady et al., 2016) observed that the betacyanin extraction process in red dragon fruit peel (*Hylocereus polyrhizus*) required a longer extraction time compared to the fruit flesh. (Ramli et al., 2014) observed that if the same extraction time was used, the ethanol:water ratio of the solvent had to be increased in the peel compared to the flesh.

### 3.3 Other extraction methods

Other extraction methods have been studied in the search to improve the efficiency of conventional extraction when using enzymatic treatment with pectinases (Naderi et al., 2010), ultrasound-assisted extraction (Haq et al., 2020, Silva et al., 2020, Wang et al., 2020), β-cyclodextrin (CD)-enhanced ultrasound-assisted extraction (Tutunchi et al., 2019), microwave-assisted extraction (Ferreres et al., 2017, Melgar et al., 2019, Singh et al., 2017), and the application of pulsed electric fields (PEFs) (Jiménez-Alvarado et al., 2015, Nowacka et al., 2019) as well as the use of supercritical fluid extraction (SFE) as a safe alternative for the environment due to the lack or minimal use of solvents compared to conventional extraction (Fathordoobady et al., 2016, Nunes et al., 2015). Some of the alternative methods to conventional extraction as well as the primary process conditions are summarized in Table 3, again indicating that the nature of the sample will determine the conditions, time and intensity of the process as well as the type of solvent and the appropriate R_L/S_. However, the high cost involved in some of these extraction alternatives compared to traditional methods undoubtedly still needs to be considered and valued (Zin et al., 2020a).

**Table 3:** Summary of some of the optimal or suggested conditions for the extraction of betalains by non-conventional extraction methods from natural sources.

**Extraction method**	**Sample**	**Compound(s)**	**R_L/S_**	**Solvent**	**Solvents ratio (%)**	**Temperature**	**Time**	**Other condition**	**Reference**
Enzimatic treatment	*Hylocereus polyrhizus* fruit	betanin, isobetanin, phylocactin, hylocerenin, isophyllocactin, and isohylocerenin	1 mL/g	water (acidified with citric acid, pH 4)	100	40 °C	120 min	stirring speed of 250 rpm	Naderi et al., 2010
Ultrasound-assisted	Colored quinoa (*Chenopodium quinoa* Willd) hulls	betacyanins	100 mL/g	water	100		9.2 s	power of 100 W, 30 kHz, 70% of amplitude, pulse of 0.6	Laqui-Vilca et al., 2018
		betaxanthins	100 mL/g	water	100		40 s	power of 100 W, 30 kHz, 90% of amplitude, pulse of 0.7	Laqui-Vilca et al., 2018
	Grown red and golden beets (*Beta vulgaris* L.)	betacyanins and betaxanthins	2 mL/g	methanol	100		60 min		Wang et al., 2020
	Red beet (*Beta vulgaris* L.)	betacyanins and betaxanthins	5 mL/g	ethanol:water (acidified with acetic acid, 0.5%)	30:70–45:55	55 °C	15 min	37 kHz. After sonication, stirring at 320 rpm for 43 min at 40 °C	Haq et al., 2020
		betacyanins and betaxanthins	75 mL/g	water	100	30 °C	30 min	power of 83 W	Silva et al., 2020
		betacyanins	25 mL/g	ethanol:water	25:75	52 °C	90 min	power of 165 W, 25 kHz	da Silva et al., 2018
		betaxanthins	25 mL/g	ethanol:water	25:75	37 °C	90 min	power of 165 W, 25 kHz	da Silva et al., 2018
		betacyanins and betaxanthins	15 mL/g	water (pH 2.5)		50 °C	10 min		Kushwaha et al., 2018
		betacyanins and betaxanthins	19 mL/g	water	100	53 °C	35 min	power of 89 W	Maran & Priya, 2016
	Red dragon fruit (*Hylocereus polyrhizus*) flesh	betacyanin	25 mL/g	water	100	25 °C	30 min	50 kHz	Ramli et al., 2014
	Red pitaya (*Stenocereus stellatus*)	betacyanins and betaxanthins	≈2 mL/g	water	100	20 °C	15 min	40 kHz. After sonication, stirring at 3200 rpm	Pérez-Loredo et al., 2017
	Opuntia engelmannii fruit peel	betacyanins	200 mL/g	methanol:water (pH 7)	17:83	33.9 °C	1.2 min	40 kHz, stirring speed of 200 rpm	Melgar et al., 2019
β-CD-enhanced ultrasound assisted	Red beets (*Beta vulgaris* L)	betanin	10 mL/g	water:β-Ciclodextrin (β-CD)	95:5		30 min	28 kHz, 80 W. Prior to ultrasound treatment, the sample solution was homogenized for 180 min.	Tutunchi et al., 2019
Microwave assissted	Dragon fruit (*Hylocereus polyzhirus*) peel	betalains	25 mL/g	water	100	35°C	8 min	microwave power of 100 W	Thirugnanasambandham & Sivakumar, 2015
	Red beetroot (*Beta vulgaris* L.)	betacyanins (betanin)	250 mL/g	ethanol:water (acidified with ascorbic acid, 0.04 mol/L)	50:50		1.17 min/1.7 min	microwave power of 400 W; duty cycle of 100%	Cardoso-Ugarte et al., 2014
		betaxanthins	250 mL/g	ethanol:water (acidified with ascorbic acid, 0.04 mol/L)	50:50		2.7 min/1.8 min	microwave power of 400 W; duty cycle of 100%	(Cardoso-Ugarte et al., 2014
	Red beetroot (*Beta vulgaris* L.) peel	betacyanins (betanin)	5 mL/g	water (acidified with citric acid, pH 5.2)	100		0.95 min	microwave power of 224.61 W	Singh et al., 2017
		betacyanins (betanin)	5 mL/g	ethanol	100		1.25 min	microwave power of 384.25 W	Singh et al., 2017
		betacyanins and betaxanthins	(4:1, 2:1, 2:1, and 1.5:1)	water	100		12 min (4 times of 3 min)	microwave power of 450 W	Slavov et al., 2013
	*Opuntia engelmannii* fruit peel	betacyanins	49 mL/g	methanol:water (pH 7)	55:45	25°C	8.8 min	microwave power of 400 W	Melgar et al., 2019
	White-fleshed red pitaya (*Hylocereus undatus*)	betacyanins	150 mL/g	water	100	49.33°C	5 min	microwave power of 600 W	Ferreres et al., 2017
	Yellow pitaya (*Hylocereus megalanthus)*	betacyanins	150 mL/g	water	100	49.33°C	5 min	microwave power of 600 W	Ferreres et al., 2017
Pulsed electric field	Red beet (*Beta vulgaris* L.)	betanin and vulgaxanthin	100 mL/g	phosphate buffer, pH 6.5	100			20 μs pulses of electric field at 4.38 kV cm^−1^ of strength, Energy of 4.86 kJ/kg.	Nowacka et al., 2019
		betalains	20 mL/g	water	100			100 μs pulses with electric field strengthat 1 kV cm^−1^ of strength.	Loginova et al., 2011
High-Pulsed Electric Fields (HPEF)	Prickly Pear (*Opuntia* spp.) fruits	betacyanins and betaxanthins					10 min	8 kV cm^−1^, repetition rate of 5 Hz.	Jiménez-Alvarado et al., 2015
﻿High Pressure Carbon Dioxide (HPCD)	Cactus pears (*Opuntia* spp.) fruit	betacyanins and betaxanthins		sample + water (acidified with citric acid, pH 5):CO_2_ pressurized	20:80	40°C	30 min	high pressure CO_2_ pre-treatment of dried sample pre-heated to 55°C, CO_2_ at 375 bar for 60 min	Nunes et al., 2015
Supercritical Fluid Extraction (SFE)	*Hylocereus polyrhizus* flesh and peel	betacyanins		co-solvent (ethanol:water 10:90):CO_2_ pressurized	90:10	50°C	90 min	pressure of 25 Mpa	Fathordoobady et al., 2016

RL/S mean: Ratio liquid (solvent)/solid.

## 4 Analysis of betalains

The color of betalains is due to their structural chromophore group (Fig. 2), which has allowed for UV–Vis spectroscopy to be the most widely used analytical technique for the quantitative identification of their two structural groups (betacyanins and betaxanthins) in natural sources. The measurement of the maximum absorption in the visible region at 480 nm is used for the quantification of betaxanthins and at 535–538 nm for betacyanins. Through a mathematical calculation that considers the dilution factor, the molecular weight of betalains (308 g/mol for betaxanthin, 550 g/mol for betanin, and 726.6 g/mol for amaranthin) and its extinction coefficient (*ε*) (48,000 L/mol·cm for betaxanthin, 60,000 L/mol·cm for betanin, and 56,600 L/mol·cm for amaranthin, in H_2_O), the total proximal content of betalain compounds can be determined (Haq et al., 2020, Kushwaha et al., 2018, Silva et al., 2020, Tutunchi et al., 2019, Yap et al., 2019, Zin et al., 2020a). However, the system of conjugated double bonds gives betalains the property of fluorescence absorption/emission, for a maximum excitation at 320–475 nm, corresponding to blue light, and emission at 506–660 nm correspondingly to green light (Slimen et al., 2017). Fourier transform infrared (FTIR) analysis has also been one of the tools used to evaluate the presence of betalains, although indirectly, since through this tool, it is only possible to identify the amine group of nitrogen (N–H) that would indicate the possible presence of these pigments. Some of the betalains signals studied by FTIR are located at 1651 cm^−1,^ which is associated with the presence of the carbonyl group (C = O) in stretching mode associated with the amide bond; 1641 cm^−1^ is related to the N–H bend of the 1° amine group, the band at 1050 cm^−1^ represents the C–N stretching of the amine, and the measurement at 718 cm^−1^ confirms the presence of the amine group (N–H) (Kumar et al., 2017, Singh et al., 2017, Tutunchi et al., 2019).

Through tools such as liquid chromatography (LC), in the high-pressure liquid chromatography (HPLC) modality (Ferreres et al., 2017, García-Cruz et al., 2017, Wang et al., 2020), ultra-performance liquid chromatography (UPLC) (Melgar et al., 2019), or ultra-high-pressure liquid chromatography (UHPLC) (Cejudo-Bastante et al., 2014, Wang et al., 2020) in reverse-phase (RP), the separation of betalain mixtures has been very effective. Some of the solvents used as mobile phases to separate betalains by LC are acetonitrile–water (Fathordoobady et al., 2016, Ferreres et al., 2017, García-Cruz et al., 2017, Sawicki et al., 2016, Sawicki et al., 2017, Wang et al., 2020), methanol–water (Cejudo-Bastante et al., 2014, Slavov et al., 2013), and acetonitrile buffer of KH_2_PO_4_ (pH 2.74) (Sanchez-Gonzalez et al., 2013) in different proportions, which are applied by eluting in isocratic mode or modifying the gradient concentration of the phase. The acidification of the mobile phase with 0.012–1.0% formic acid (Fathordoobady et al., 2016, Ferreres et al., 2017, García-Cruz et al., 2017, Sawicki et al., 2016, Sawicki et al., 2017, Wang et al., 2020) or with trifluoroacetic acid (TFA) 0.05% (Naderi et al., 2010) is common in this analysis to maintain the stability of the betalains structure during the process. The coupling of LC to detectors, such as UV–Vis spectroscopy, diode array detection (DAD), and mass spectrometry (MS), has enabled the qualitative and quantitative analysis applied to the characterization of the betalain pigments profile from several natural sources, stability studies, and evaluations of the extraction efficiencies of specific betalains of particular interest. In detection by UV–Vis spectroscopy, monitoring at a wavelength of 480 or 540 nm is recommended (Melgar et al., 2019, Naderi et al., 2010, Slavov et al., 2013). For DAD, using a monitoring window at 477–484 and 535 nm is recommended (Fathordoobady et al., 2016, Ferreres et al., 2017, García-Cruz et al., 2017, Melgar et al., 2019, Wang et al., 2020). In detection by MS by time-of-flight (TOF) applying electrospray ionization (ESI) (Fathordoobady et al., 2016, Ferreres et al., 2017, García-Cruz et al., 2017, Melgar et al., 2019, Sawicki et al., 2016, Wang et al., 2020) or by tandem mass spectrometry (MS/MS) (Sawicki et al., 2017), operating the electrospray ionization source in positive mode is suggested.

## 5 Stability and encapsulation of betalains

Betalains are approved as colorants by the European Union and by the Food and Drug Administration and have been used in several food products (Khan, 2016), since they are also considered a valuable antioxidant resource, so their consumption could enhance protection against free radicals (Wybraniec, 2005). However, its use has been reduced due to its low stability, since its properties and coloring power are affected by several factors, which have been widely studied in various research studies (Table 4). In this regard, the temperature is one of the factors that has the greatest effect on the structure of betalains. An increase in temperature results in an increase in the degradation of betalains (Güneşer, 2016, Kayın et al., 2019, Laqui-Vilca et al., 2018, Prieto-Santiago et al., 2020), the structure of which is modified due to hydrolysis, isomerization, dehydrogenation, deglycosylation, and decarboxylation processes (Herbach et al., 2006). High temperatures cause the decarboxylation of betanin, generating neobetanin, which produces a color change due to the formation of an aglycone with less stability (Herbach et al., 2006, Reshmi et al., 2012). The thermal degradation of betalains has also been reported to produce mono-, di-, and tricarboxylic betacyanins (Wybraniec, 2005). The effect of the temperature on betalains degradation is influenced by the intensity of heating, the presence of oxygen, the concentration of pigments present, pressure, ultrasound, and other factors (dos Santos et al., 2018, Güneşer, 2016, Laqui-Vilca et al., 2018, Prieto-Santiago et al., 2020). Betalamic acid is susceptible to isomerization due to the temperature effect. The hydrolysis of betanin leads to the breakdown of the molecule, generating betalamic acid and *cyclo*-dopa-5-O-beta-glucoside, for an imminent decrease in coloration (Herbach et al., 2006). Oxygen is another critical factor in the degradation of betalains, especially since its effect has been related to joint degradation with other factors, such as the presence of light and temperature (Barba-Espin et al., 2018, von Elbe and Attoe, 1985). Betalains show stability at a pH of 4–6 and at a temperature of 4 °C; as a result, betalains are degraded at pH values outside this range and change color depending on the pH of the sample. Betalains below pH 3 present a violet color, and betalains at a pH greater than 7 exhibit a blue color (Wootton-Beard & Ryan, 2011). At pH >7, betanin is degraded by the hydrolysis of aldimine bonds, producing ferulic acid with an amine group (Khan, 2016). The presence of specific metals has also been reported to affect betalains degradation (Khan & Giridhar, 2014), so to reduce the effect of metal ions, chelating agents have been used, such as ascorbic acid or citric acid (Stintzing & Carle, 2008), which have been known to remove O_2_ from the solution and reduce the polarity at the N-1 position of betalains, which is susceptible to nucleophilic attack by water (Herbach et al., 2006). Betalains are easily degraded by light; this degradation is due to the absorption of UV light (Chhikara et al., 2019), and the degree of the effect depends on the light intensity, the presence or absence of oxygen, and the concentration and reactivity of betalains (Kayın et al., 2019). However, infrared light reportedly favors the accumulation of betacyanins in red beet roots (Shin et al., 2003). It has also been reported that water activity is a factor that must be controlled in products containing betalains, since high water activities potentiate betalains degradation, while low water activities improve their stability (Chhikara et al., 2019). There is also another series of processes, such as microwaving, boiling, roasting, vacuum, high pressure, ultrasound fermentation, pasteurization, and the use of additives, that have been shown to affect the stability of betalains in different products (Czyzowska et al., 2006, Laqui-Vilca et al., 2018, Moßhammer et al., 2007, Ravichandran et al., 2013, Sawicki et al., 2019). All these factors affect the structure of betalains in some way, which is reflected in a change in the color parameters (Güneşer, 2016, Moßhammer et al., 2007, Prieto-Santiago et al., 2020), so its control must be considered for use as a colorant and the development and incorporation into food products since the presence of all these factors limit their application in food, which is why several techniques have been used for their preservation. The encapsulation of betalains can help maintain their stability, increase their useful life, and improve their handling. Several factors have been reported that affect the retention of encapsulated betalains, such as the type and concentration of the wall material, the encapsulation technique, and the encapsulation conditions, among other factors. Among the wall materials, maltodextrin alone or in combination with other biopolymers is the polysaccharide that has been used most frequently for the encapsulation of betalains (Castro-Enríquez et al., 2020). However, maltodextrins have high solubility and hygroscopicity at high water activities (Prieto-Santiago et al., 2020), which is why materials such as gum arabic and whey protein concentrate, alginate, lecithin, and others have been used, which have been shown to delay or maintain the stability of the encapsulated betalains in addition to maintaining the integrity of the capsule at low water activities (Pitalua et al., 2010). Some studies have shown that the use of binary and ternary blends of polymers as wall materials produces higher retention and less degradation of betalains than using a single polymer, which is reflected in a higher encapsulation efficiency and greater retention of betalains during storage, and that the use of a second polymer generally increases the viscosity of the solution, leading to the formation of a thicker protective wall, which restricts the movement of betalains (Hogan et al., 2001).

**Table 4:** Effect of processing factors on the stability of betalains obtained from different food sources.

**Process**	**Conditions**	**Products**	**Main Findings**	**Reference**
Storage temperature and light	25, 35 and 45 °C and light with/without aluminum foil	Red beet juice	Degradation of betalains, change in total phenols and color	Kayın et al., 2019
Heating	70–90 °C	Beet root	Degradation of betalains and color parameters.	Güneşer, 2016
Heating	autoclave (120 °C) for 10, 20, 30, 40, 50, and 60 min	Beetroot juice, beetroot puree and whole peeled beetroots	Degradation of betalains and color parameters.	Prieto-Santiago et al., 2020
Thermal stability and ultrasound treatment	0–80 °C	Colored quinoa (*Chenopodium quinoa* Willd) hulls	Thermal stability was similar to that of betalains from beetroot	Laqui-Vilca et al., 2018
High pressure processing (HPP) and high temperature short time (HTST) thermal treatment	HPP was applied at 000 bar for 10, 20 and 30 min and HTST treatment was applied at 75.7 °C for 80 s, 81.1 °C for 100 s and 85.7 °C for 120 s	Red beet stalks	HPP treatment did not show any improvement in the betalain stability. HTST was considered the most suitable to maintain betalain stability from red beet.	dos Santos et al., 2018
Presence of metals and ascorbic acid	Inorganic Se^4+^, Zn^2+,^ and Cu^2+^ metal with/without ascorbic acid	Berry juice	Ascorbic acid protected the pigments from metal-induced bleaching	Khan & Giridhar, 2014
Technological processes	Microwaving, boiling, roasting and vacuuming	Red beet	Vacuum and microwave produces increases in betalains, while boiling and roasting produces a decrease	Ravichandran et al., 2013
Lactic acid fermentation	Three probiotic bacteria and three infant intestinal microbiota of *Lactobacillus*	Red beet juice	Lactic acid fermentation influenced color parameters	Czyzowska et al., 2006
Food aditives And pH	Ascorbic, isoascorbic, and citric acid at pH 4 and 6	Yellow-orange cactus pear	Pigment stability and color characteristics depended on type and concentration of the respective additive as well as on pH conditions.	Moßhammer et al., 2007
Technological processes and *in vitro* digestion	Boiling, fermentation and microwave vacuuming treatment	Red beetroot products	Technological processes reduced the content of betalain by 42–70% in the obtained products. The contribution of betalains released from red beet products after *in vitro* digestion was detected within the range of 0.001–0.10%.	Sawicki et al., 2017

Notably, several betalains encapsulation techniques have been used, such as spray drying, lyophilization, coacervation, emulsion, ionic gelation, and sonication hydration. Spray drying turns out to be the most used method, primarily due to its low cost and the availability of equipment (Castro-Enríquez et al., 2020). The process has a high encapsulation efficiency, and the product obtained by this method is a highly manageable and versatile powder. However, for the high inlet temperatures used in the drying process, process yields may be less than 70%, and the high storage temperatures cause the degradation of the pigments encapsulated by this method (Chranioti et al., 2015; Soto-Castro et al., 2019). The primary problem with the powders obtained by spray drying is associated with the structural instability of the capsule or properties of the powder due to its high hygroscopicity that leads to a greater exposure of the betalains to environments with higher water activity and a greater exposure to oxygen, affecting their stability. Given this consideration, it is pertinent to deepen studies on new wall materials or mixtures thereof that offer these characteristics in the final powder. Another alternative encapsulation method, freeze-drying, has improved the stability of encapsulated betalains compared to spray drying (Ravichandran et al., 2014), but it is always recommended to consider the hygroscopicity of the system to guarantee its integrity and the stability of the pigment. Encapsulation in nanoliposomes using lecithin has protected the stability of betalains after ingestion *in vitro* (Amjadi et al., 2019)*,* which implies that this type of system can favor the bioavailability of encapsulated betalains. The drawback of this liposomal system is that betalains have shown degradation problems during storage when they were incorporated in matrices such as gummy candies (Amjadi et al., 2018). However, the problem is possibly more associated with the composition and characteristics of the model matrix under study, such as its high hygroscopicity and water activity, which could participate in the oxidation of nanoliposomes. Ionic gelation is a technique that has effectively protected this group of pigments during storage (Otálora et al., 2019), while gelation using sodium alginate exhibited high encapsulation efficiency (Orozco-Villafuerte et al., 2019). However, more studies are still required to show the behavior and stability of this type of system under different storage conditions and in application matrices. Lastly, the encapsulation of betalains in emulsion systems has shown high degradation sensitivity influenced by temperature (Pagano et al., 2018), but despite these characteristics, the use of these emulsions as an intermediate product to obtain a powder by means of techniques such as spray drying or lyophilization to improve its stability or diversify its applicability should not be ruled out.

## 6 Applications of betalains as an additive in food

In the search for new sources of natural additives for use in food, numerous studies have evaluated the potential use of betalains as colorants, antioxidants, and antimicrobials. Attia et al. (2013) evaluated the effect of incorporating red beet extract as a colorant in jelly and ice sherbets for its sensory properties, observing that the general acceptability of the products is dependent on the concentration of added betalains and on properties comparable to those of a synthetic red colorant. Betalains have also been incorporated as colorants for ice cream, and they improve the acceptability of the product and have good color stability for 180 days under storage at −20 °C (Kumar et al., 2015, Roriz et al., 2018). Khan et al., (2015) incorporated betalains from berries (*Rivina humilis*) as a colorant for fruit spread and banana juice, and they observed that the stability of betalains in the fruit spread was not greater than 40% after six months storage at 5 °C. In the beverage, the proposed colorant was not viable due to the total loss of betalamic color during the pasteurization process. Güneşer (2016) observed that the betalains in beet roots could present moderate stability in response to thermal treatments (70–140 min, at 70–80 °C) when they are added as a colorant to cow milk. Gengatharan et al. (2016) evaluated the effect of pasteurization (30 min at 63 °C) on the stability of betalains from red pitahaya (*Hylocereus polyrhizus*) and red beet (*Beta vulgaris*; E-162) when added as a colorant to simulate a strawberry color in cow milk. The results showed that the stability and acceptability of the color were dependent on the betalains profile of the source of origin, indicating that the betalains in red pitahaya were more stable in response to the pasteurization process and 7 days of storage at 4 °C, and they showed a higher acceptability score color compared to E-162. Rodríguez-Sánchez et al. (2017) incorporated betaxanthins from yellow pitaya (*S. pruinosus*) fruit as a coloring for drinks and jelly gummies. They observed that the greatest betaxanthin stability was achieved when the product was stored at low temperatures and under dark conditions. In addition, they observed that these pigments were more stable in the gummies because of the food matrix (a protective effect was conferred by their interactions with proteins) and their low water activity. Kumar et al. (2020) observed that betalains from *Basella rubra* can be used as a colorant for banana spread with a stability of 95% after one year of storage at 5 °C, for an intermediate moisture food (making it a gel-like product) with a stability of 60% after two months, and for juices, bananas, and lemons, with a stability of 58% and 76%, respectively, after three months. In all cases, the proposed dye inhibited microbial growth and showed good sensory acceptability in the product. The results of these studies seem to indicate that the use of betalains as a colorant in food may be highly viable after considering three critical factors: 1) the betalains profile that constitutes the proposed natural colorant; 2) the composition of the food matrix (lower water activity, higher acidity, and presence of proteins favor color stability); and 3) the food storage conditions (products stored at −20–4 °C and protected from light are the best candidates).

The use of betalains as natural antioxidants has also been studied in several foods. Attia et al. (2013) studied the antioxidant effect of red beet roots in corn oil after seven days of storage at 60 °C, observing a decrease in the peroxide index with values similar to those obtained with BHT. Coria-Cayupán & Nazareno (2015) evaluated the protective effect of betalains from cactus pear fruits that were incorporated as natural pigments in dairy products (yogurt and cream), observing an inhibition of oxidative damage greater than 80% in yogurt and 50% in cream during the oxidation of the systems without the added pigments. da Silva et al. (2019) evaluated the lipid oxidation inhibition capacity of betanin when incorporated as an antioxidant in pork meat with results similar to those obtained by adding synthetic antioxidants such as BHA and BHT up to 6 days of storage to 4 °C. These results show that the use of betalains as antioxidant agents seems to meet the growing demand for increasingly natural foods by consumers; however, due to their pigmentation characteristics, the sensory impact on the food into which they are incorporated should not be disregarded.

## 7 New trends in applying betalains in the food industry

Faced with the demand to generate strategies that improve the shelf life of food, the monitoring of product quality in real time, the minimum use of synthetic preservatives, and the reduction of negative impacts on the environment, the development of new smart packaging based on biopolymers and natural extracts has increased in the food industry (Kanatt, 2020). The pH-sensitive property of betacyanins has been used in the development of smart films with potential applications in food packaging. Jamróz et al., (2019) observed that an extract rich in betalains from beet roots in furcellaran films changed from red to green when the films were exposed to ammonia. The developed film was applied as packaging to monitor the deterioration of fish fillets stored at 2 °C; however, the film's color change was not effective enough to inform trained panelists of the deterioration of the food. Under the same principle, Qin et al. (2020) incorporated an extract containing betalains from red pitaya in starch/polyvinyl alcohol films, yielding a film with antioxidant and antimicrobial properties that was successful as an intelligent packaging material to monitor the freshness of shrimp and had the potential to monitor the freshness of protein-rich animal foods. Similar results were observed by Hu et al. (2020) when incorporating amaranth betalains in a quaternary ammonium chitosan/fish gelatin film, yielding a functional film with improved antioxidant and antimicrobial properties against pathogens in food. The film also exhibited the ability to change color with pH sensitivity under alkaline conditions, which allowed its feasibility to be evaluated as a smart packaging material for monitoring the freshness of shrimp. Additionally, the effectiveness of the film's color change can be negatively affected by a higher content of betalains in the formulation. Lastly, Yao et al. (2020) developed antioxidant, antimicrobial and ammonia-sensitive films based on quaternary ammonium chitosan/polyvinyl alcohol with betalain extracts from cactus pears (*Opuntia ficus-indica*) and applied them as intelligent packaging materials that change color (from purple to orange) when shrimp lose their freshness. This finding indicates that some sources of betalains may have a place in the food industry not only as additive colorants, antioxidants, or antimicrobials but also because their participation is projected to become significant for the innovation and development of intelligent materials for the packaging of seafood industry products.

## 8 Conclusions

Each of the wall materials and encapsulation techniques used in the different investigations has advantages and disadvantages that must be considered during the development of microcapsules to obtain betalains with maximum stability and that affect their coloring properties to a lesser extent. Betalains have shown their potential as colorants, antioxidants, and antimicrobials in food matrices. Therefore, develop of intelligent and/or active materials for food packaging is very promising for the application and use of the properties of betalains in other fields of the food industry in the quest to extend shelf life, increase food safety, and reduce negative environmental impacts.

## Declaration of Competing Interest

The authors declare that they have no known competing financial interests or personal relationships that could have appeared to influence the work reported in this paper.
